# Factors Associated with Stress among Healthcare Personnel after COVID-19 in Northeast Thailand: A Cross-sectional Study

**DOI:** 10.2174/0117450179327231240924054645

**Published:** 2024-10-08

**Authors:** Pornpimon Chupanich, Pratoomrat Aotprapai, Sakda Seesophon, Pokkamol Laoraksawong

**Affiliations:** 1 Department of Public Health Administration, Health Promotion, and Nutrition, Faculty of Public Health, Khon Kaen University, Khon Kaen, Thailand; 2 Faculty of Public Health, Khon Kaen University, Khon Kaen, Thailand; 3 Department of Occupational Safety and Environmental Health, Faculty of Public Health, Khon Kaen University, Khon Kaen, Thailand

**Keywords:** Prevalence, Factors, Stress, Healthcare personnel, COVID-19, Thailand

## Abstract

**Background:**

The coronavirus disease (COVID-19) has affected people psychologically worldwide, particularly healthcare personnel. Even though the COVID-19 pandemic situation has eased, healthcare personnel must still perform their duties, which has resulted in psychological impacts, particularly stress.

**Objectives:**

This study aimed to examine the risk factors associated with stress among healthcare personnel post-COVID-19 pandemic in northeast Thailand.

**Methods:**

A cross-sectional analytic design was conducted from January to April 2023. One thousand and three hundred healthcare workers were selected from primary, secondary, and tertiary hospitals across 16 districts within Chaiyaphum province. The questionnaires were used to collect data, and the stress test 5 (ST-5) questionnaire was used to investigate stress among healthcare personnel.

**Results:**

The overall stress rate for healthcare workers was 15.47%, including very severe (8.85%) and severe (6.62%). The factors associated with stress consisted of work position, environment of work, personal life such as education level and income, and responsibility for taking care of family members, in addition to experiencing quarantine from COVID-19 were more likely to have a high risk of stress problems among healthcare workers.

**Conclusion:**

This result highlighted that the mental health of personnel should be in critical situations, and those found severely afflicted should undergo professional care. To prevent psychological issues, particularly stress, health organizations should be concerned with strong organizational management, which includes supporting bonuses and providing high-quality personal protective equipment (PPE) to healthcare staff.

## 1 INTRODUCTION

COVID-19 was discovered in 2019 in Wuhan, China. After that, COVID-19 spread rapidly worldwide and became a global health threat. Globally, COVID-19 cases were confirmed over 774 million and over seven million deaths have been reported as of January 19^th^, 2024 [1]. In Thailand, the total number of COVID-19 cases reached over 4 million confirmed cases and 34,521 deaths as of January 7^th^, 2024 [2]. Despite easing the COVID-19 pandemic, healthcare personnel must still perform their duties such as monitoring, controlling disease outbreaks, recommending, following up on patients' symptoms, preparing epidemiological, statistical reports, *etc*. Beyond the situation of infection, COVID-19 also affects the mental health of healthcare personnel, especially stress.

Stress is a psychological and physiological response to undesirable experiences [3]. The impact of stress on healthcare personnel is important. Stress can cause biological reactions in the body, such as nervous system functions, immune system functions, and cardiovascular system functions that trigger or aggravate factors for many diseases and pathological conditions [4]. The research examining the effects of the COVID-19 pandemic on the mental health of healthcare professionals commenced at the onset of the pandemic across numerous nations [5]. A systematic review and meta-analysis from 18 countries in the Asia region showed that the prevalence of stress was 31.72% (95% CI: 21.2 – 42.18%) [6] and the high prevalence was found in frontline healthcare providers, females, and nurses [6-11]. In Thailand, the prevalence of perceived stress among healthcare workers ranged from 23.30% to 41.97% during the COVID-19 outbreak [12, 13]. Previous studies showed various stress-related factors such as age, gender, work experience, work hours, family factors, and caring for COVID-19 patients [14-16].

However, the prevalence and risk factors for stress among healthcare personnel before and after COVID-19 differed. Therefore, this study aimed to examine the risk factors related to stress among healthcare personnel post-COVID-19 pandemic in northeast Thailand.

## 2 MATERIALS AND METHODS

### 2.1 Population and Samples

This cross-sectional analytic study was carried out from January to April 2023. In this study, the sample size was determined using multiple logistic regression [17] as follows: the proportion of stress in men (*p*_1_) and women (*p*_2_) of healthcare personnel were 0.27 and 0.17, respectively [18]. The total proportion (*P*) of stress was 0.5. The predetermined sample size for this investigation comprised 1,076 healthcare personnel. Accounting for an anticipated reduction of approximately 20% due to questionnaire non-responses, the researchers adjusted the initial sample size to 1,300 healthcare personnel. Utilizing a simple random sampling approach, participants were selected from primary, secondary, and tertiary hospitals across 16 districts within Chaiyaphum province, representing diverse healthcare disciplines, including physicians, dentists, medical technologists, nurses, pharmacists, public health technical officers, and public health officers. Inclusion criteria stipulated participants' tenure of more than 1 year within primary, secondary, or tertiary hospital settings and an age of ≥ 20 years. Exclusion criteria encompassed individuals diagnosed with mental health disorders.

### 2.2 Research Instruments

The questionnaire consisted of 5 sections (27 items), including 1) personal characteristics, 2) job-related characteristics, 3) motivating factors, 4) organizing administration, and 5) stress test 5 (ST-5) questionnaire. The first to fourth sections (22 items) incorporate open-ended and closed-ended questions developed based on relevant theories and prior research. Section 5 utilized the ST-5 questionnaire, a tool developed by the Department of Mental Health, Ministry of Public Health in Thailand, for assessing stress levels [19]. The ST-5 has demonstrated a sensitivity of 92.7% and specificity of 90.7%, indicating its effectiveness as a screening tool [20]. The stress scores were interpreted as 4 levels, including mild (0-4 scores), moderate (5-7 scores), severe (8-9 scores), and very severe (10-15 scores).

### 2.3 Data Collection

The researchers wrote to the administrators of one of the chosen hospitals to request permission and to outline the purpose and methodology of the study. The 1,300 samples from 16 districts in the province of Chaiyaphum were randomized. Google Forms was used to create an online survey for collecting data. On the first page of the form, participants were informed that their participation in the study was completely optional and that the aims had been properly disclosed to them. Before the collection of data and samples, participants were entirely voluntary and written informed consent was obtained from all participants.

### 2.4 Data Analysis

The STATA program version 18 (College Station, TX: StataCorp LLC) under license from Khon Kaen University was used to analyze the data. Descriptive statistics were presented as percentages, mean values, standard deviations, and minimum-maximum values. Inferential statistical analysis used simple and multinomial logistic regression analysis. All variables with a P-value less than 0.25 in the simple logistic regression analysis were selected for the multinomial logistic regression analysis to adjust for possible confounders. The results were presented by Crude Odds Ratios (Crude OR) and Adjusted Odds Ratios (Adj. OR) along with a 95% Confidence Interval (95%CI) to establish statistical significance at the 0.05 level.

## 3 RESULTS

### 3.1 Demographic Characteristics

In a total of 1,300 participants, most of the healthcare personnel were physicians and nurses (71.85%), female (69.38%), aged 30-39 years old (39.77%), married (69.69%), had an education of bachelor's degree or higher (83.69%), healthy (90.0%), and income more than 809.0$ (45.46%) (according to the USD-THB exchange rate as of April 29^th^, 2024). The majority of healthcare personnel worked at a secondary or tertiary hospital (79.0%), worked more than 40 hours/week (70.0%), and more than half of the healthcare personnel had work experience of more than 10 years (53.77%). Most of the healthcare personnel were living with the elderly (75.08%), had ≥ 5 family members, and three hundred and seventy-six (28.92%) lived with a grandchild who was aged < 5 years old. Most of the healthcare personnel had been infected with COVID-19 (83.77%) and had quarantined experience because they had a risk of being infected with COVID-19 (84.92%) (Table **1**). For organizational administration, most of the participants 1,249 (96.08%) had supported bonuses or allowances, while 858 (66.0%) were satisfied with a bonus or allowance. Eight hundred and forty-two (64.77%) healthcare personnel had an appropriate shift schedule. Additionally, 878 (67.54%) of healthcare personnel received inadequate personal protective equipment (PPE), but almost all PPE, 1,206 (92.77%) were of good quality (Table **1**).

### 3.2 Prevalence of Stress and Factors associated with Stress in Healthcare Personnel

The prevalence of stress among healthcare personnel post-COVID-19 pandemic was as follows: 115 (8.85%) were very severe, 86 (6.62%) were severe, 544 (41.85%) were moderate, and 555 (42.69%) were mild (Table **2**).

A total of 22 covariables were considered for backward stepwise regression analysis, of which 16 variables were selected based on their Crude OR at a 25% level of significance. Before developing the multinomial logistic regression, variables were assessed for collinearity and first-order effect modifier (Table **2**). For multiple multinomial logistic regression, the physicians and nurses were 36% more likely to have very severe stress as compared to mild stress levels (Adj. OR=1.36, 95%CI: 1.04 to 1.78, P-value = 0.026). The healthcare personnel who were married were 36% more likely to have severe stress as compared to mild stress levels (Adj. OR=1.36, 95%CI: 1.03 to 1.79, P-value = 0.03). Healthcare personnel who graduated with a bachelor's degree or higher were 88% more likely to experience severe stress as compared to those who graduated with a lower bachelor's degree (Adj. OR=1.88, 95%CI: 1.25 to 2.80, P-value = 0.002). Moreover, participants who worked at a secondary or tertiary hospital were 89% more likely to have severe stress as compared to healthcare personnel in a primary hospital (Adj. OR=1.89, 95%CI: 1.44 to 2.49, P-value <0.001), whereas healthcare personnel who had income > 809.0$ had 36% less chance of severe stress (Adj. OR=0.64, 95%CI: 0.41 to 0.99, P-value < 0.001). Additionally, healthcare personnel who had a grandchild aged < 5 years were 89% more likely to have severe stress than those who had a grandchild aged > 5 years (Adj. OR=1.89, 95%CI: 1.48 to 2.41; P-value < 0.001), and healthcare personnel who had a family member with Non-Communicable Diseases (NCDs) were 51% more likely to have severe stress than those who had no family member with NCDs (Adj. OR=1.51, 95%CI: 1.21 to 1.89; P-value < 0.001). Moreover, participants who had experienced quarantine were 52% more likely to have severe stress than those who had no experience with quarantine (Adj. OR=1.52, 95%CI: 1.11 to 2.07, P-value = 0.009) (Table **3**).

For organizing administration, the healthcare personnel who were not supported by bonuses or allowances were 2.51 times more likely to have severe stress (Adj. OR=2.51, 95%CI: 1.42 to 4.46, P-value = 0.002). Healthcare personnel who used low-quality PPEs were 2.56 times more likely to have severe stress (Adj. OR=2.56, 95%CI: 1.71 to 3.82, P-value = <0.001). Furthermore, participants who worked overload were 40% more likely to have severe stress (Adj. OR=1.40, 95%CI: 1.11 to 1.78, P-value = 0.005) (Table **3**).

## 4 DISCUSSION

After the COVID-19 pandemic, many healthcare personnel are threatened with being infected and must handle the extraordinary workload. So, their mental health would be affected even more compared to the general population due to their daily experience, especially stress. Overall stress rates for healthcare workers in the predominantly rural province of Thailand are rather high, with about 42% for moderate, 7% for severe, and 9% for very severe. Occupational stress has been recognized as one of the major occupational health hazards, particularly personnel in the medical field who usually face a more stressful environment than personnel in other industries [21]. The Thai healthcare personnel, like many other healthcare personnel around the world, have had to deal with the consequences of the COVID-19 virus, resulting in stress. Previous studies showed that the high prevalence of stress ranged from 44.0% to 100.0% among healthcare workers worldwide, especially in China, and India [8, 22-26]. Moreover, a systematic review showed that the prevalence of stress in Asia regions was higher than in other regions [6, 27]. The higher prevalence of stress may be attributed to the widespread uncertainty created by the ongoing pandemic, the limited availability of an effective vaccine, an increased workload, insufficient social support, and a heightened fear of transmitting the virus to family members [27-30]. In the previous study, Thailand was one of the top three countries of the seven middle-income countries in Asia with the highest stress scores (mean 21.94, SD 7.74), followed by Pakistan (mean 14.02, SD 11.53) and the Philippines (mean 10.60, SD 8.01) [31]. Healthcare personnel of all 12 health regions in Thailand had mild to extremely severe stress of 15.3% during the first wave of the COVID-19 pandemic [32]. So, the prevalence of stress in this study was higher than in the first wave of the COVID-19 pandemic in Thailand. One possible explanation is that healthcare personnel have been working extensively during outbreaks, handling patients for a prolonged period until COVID-19 became endemic. It causes an accumulation of stress in healthcare personnel. Prolonged stress leads to burnout, defined as emotional exhaustion, depersonalization, and diminished professional efficacy among healthcare personnel, hindering patient care and increasing medical errors, which can have severe consequences [33-35]. However, a comparison with the results of similar investigations is problematic since these were obtained mainly during the beginning of the disease outbreak based on the experience of 2021. This study was conducted retrospectively at the start of the year 2023. Because of the effective isolation of the country and the well-functioning public health administration and village health volunteers, the virus could be kept at bay throughout 2020. After that, the variant became less severe but more infective [36, 37]. It cannot be excluded that the magnitude of mental health stress experienced by the study participants after the epidemic faded was overestimated or underestimated.

The present study investigated factors related to stress post-COVID-19 pandemic. Physicians and nurses, married status, graduated with a bachelor's degree or higher, income, worked at a secondary or tertiary hospital, family with a child aged < 5 years, and family members had NCDs, as well as experienced quarantine from COVID-19, were associated factors with severe stress levels among healthcare personnel. Stress among healthcare personnel is multifactorial for instance, work overload, working environment, work experience, workplace conflict, inadequate resources, marital status, educational status, job satisfaction, *etc* [38]. Physicians and nurses are stressful occupations because they are associated with complex job skills, high expectations, high workloads, and excessive responsibilities, such as being closer, direct care, and prolonged contact with COVID-19 patients [39-43]. The previous study found that almost all physicians and nurses (91.60%) had mild to very high occupational stress, of which 64.71% considered high to very high stress [21]. The other studies supported this finding; physicians and nurses face high job demands, long working in rotating shifts, working for 48 hours or more per week, and lack support from other staff associated with significantly high occupational stress [44, 45]. Moreover, a previous report found that nurses had a chance 1.4 -1.6 times to be stressed [46, 47]. Therefore, there is a need to provide spiritual and emotional healthcare services to healthcare workers, especially those on the frontlines, to alleviate their psychological distress and improve their health [18, 41, 48]. Married healthcare personnel are associated with more severe stress levels than unmarried personnel. This implies that the married status can be a source of stress due to the responsibility and pressure on an individual to care for patients and worry about their family or children [49, 50]. However, previous studies showed that the support obtained from marriage or the relationship with the spouse benefits married individuals and can relieve stress at work [51-53]. Our study found that the sociodemographic factor associated with stress was income; healthcare personnel who had enough income had less chance of severe stress. The previous study reported that income was a significant factor and that healthcare providers with lower incomes were more likely to experience high levels of stress [54]. This finding is important because it highlights the need for public health policy to focus on reducing stress levels for healthcare personnel by considering appropriate remuneration for healthcare personnel. Our findings found that healthcare personnel working in a secondary or tertiary care hospital were associated with more severe stress levels than those who worked in a primary care hospital. This finding aligned with prior research indicating that healthcare workers employed in a secondary or tertiary care hospital have heightened levels of stress compared to those in primary care settings. The elevated stress levels are attributed to the larger patient caseloads and increased work demands [3, 21]. In addition, healthcare personnel with married status had to take care of children aged < 5 years in the family, and family members with NCDs were associated with severe stress levels. Families serve as a crucial support system for most individuals, especially healthcare personnel. The stress experienced by healthcare personnel is partly due to their concern for the well-being of their loved ones. Healthcare personnel 's families with children and older adult members were more stressed because they may have heightened fears of severe illness, as older adults are at higher risk for severe symptoms and greater fatality [55]. This finding could be explained by healthcare personnel having more concerns and responsibilities toward family members besides patients and routine work, as they harbor apprehensions regarding the potential infection of family members and the challenges associated with implementing strategies such as physical distancing or isolation in contexts wherein healthcare workers concurrently fulfill roles as caregivers or supporters within familial dynamics [3, 15, 46, 47, 56, 57]. Similar to the finding, the many factors contributing to stress after the pandemic of COVID-19 virus in the general population were economic, environmental, family, social, and marital statuses [3, 47, 57-59]. Additionally, supporting bonuses or allowances, inadequate PPE against the virus, and appropriate work shift allocation were related to stress. This result was consistent with previous studies that suggested appropriate workplace support is a protective factor [56, 60, 61]. Previous studies reported inappropriate shifts or long-time work related to psychological problems in healthcare workers, especially the night, afternoon, and afternoon–night rotating shifts [57, 62, 63]. Healthcare workers have less access to PPE or insufficient PPE, which is a risk factor for severe stress. This finding aligned with previous research indicating that healthcare professionals who perceive the provided PPE to be insufficient are at higher risk for psychological disorders, particularly stress [64-67]. Furthermore, this study found that older ages (≥50 years) were related to a higher risk of higher levels of stress. The older ages were concerned about COVID-19 infecting or being fatal because older adults had higher fatality risks of COVID-19 infection. This finding contrasts with previous studies that reported younger people had a higher risk of elevated stress levels [68-71]. This increased risk was attributed to younger healthcare workers possibly being less likely to have experienced such emergencies, along with their engagement with social media and the escalating economic difficulties confronting younger populations during this period [66, 72-75].

This outcome suggested that effective organizational management may contribute to healthcare personnel experiencing positive mental well-being by meeting their expectations for professional development and supporting a harmonious work-life balance. Therefore, organizations should provide essential financial and material resources and ensure appropriate working hours for their staff [15, 76]. However, it is important to note that this study has limitations. This study collected data *via* online channels, which participants answered by themselves. This might represent the bias in the results.

## CONCLUSION

The after-effects of mental health stress in the workforce of the health delivery system are hazardous not only for the individual health of the personnel but also for their work performance. They could challenge the performance of the whole system. Hopefully, the COVID-19 affair will be an extraordinary event that will not be repeated soon. In a similar situation, the source of infection should be resolved. The personnel of the health delivery system, as the immediately involved experts, should be more trained to understand the underlying reasons for the spread of infection and the new technology of treatment and vaccine production, as well as their benefits and dangers. The mental health of the personnel should be in critical situations, and those found severely afflicted should undergo professional treatment.

## Figures and Tables

**Table 1:** Characteristics of HPCs in northeast Thailand (N= 1,300).

**Factors**	**No. (%)**
**Personal Characteristics**	-
**Gender**	-
Male	398 (30.62)
Female	902 (69.38)
**Age**	-
< 30	319 (24.54)
30 - 39	517 (39.77)
40 - 49	330 (25.38)
≥ 50	134 (10.31)
**Status**	-
Single or devoted	394 (30.31)
Married	906 (69.69)
**Education**	-
Lower bachelor	212 (16.31)
Bachelor or higher	1,088 (83.69)
**Profession**	-
Other healthcare personnel	366 (28.15)
Physician and nurses	934 (71.85)
**Underlying diseases**	-
No	1,170 (90.00)
Yes	130 (10.00)
**Income**	-
< 404.50$	219 (16.85)
404.50 - 809.00$	490 7.69)
> 809.00$	591 (45.46)
Mean (SD)	28,325 (12,624)
Median (min: max)	28,000 (6500: 8,5000)
**Adequate income**	-
Inadequate	188 (14.46)
Adequate but no saving	284 (21.85)
Adequate and saving	828 (63.69)
**Workplace**	-
Primary care	273 (21.00)
Secondary or tertiary hospital	1,027 (79.00)
**Work experience**	-
< 10 years	601 (46.23)
≥ 10 years	699 (53.77)
Mean (SD)	12.07 (8.63)
Median (min: max)	10 (1: 37)
**Working hours per week**	-
≤ 40 hours per week	390 (30.00)
> 40 hours per week	910 (70.00)
Mean (SD)	48.18 (7.88)
Median (min: max)	48 (40: 96)
**Motivating factors**	-
**Number of family members**	-
< 5	434 (33.38)
≥ 5	866 (66.62)
**Median** (min: max)	5 (3: 11)
**Had a grandchild who was < 5 years old?**	-
No	924 (71.08)
Yes	376 (28.92)
**Lived with elderly**	-
No	324 (24.92)
Yes	976 (75.08)
**A family member had NCDs**	-
No	832 (64.00)
Yes	468 (36.00)
**Have you been quarantined because you had a risk of being infected with COVID-19?**	-
No	196 (15.08)
Yes	1,104 (84.92)
**Have you been infected with COVID-19?**	-
No	211 (16.23)
Yes	1,089 (83.77)
**Organizational administration**	-
**Did your organization support bonuses or allowances?**	-
No	51 (3.92)
Yes	1,249 (96.08)
**Did you get satisfactory bonuses or allowances?**	-
No	442 (34.00)
Yes	858 (66.00)
**Did your organization adequately allocate PPE to prevent COVID-19 infection?**	-
No, PPE was inadequately allocated.	878 (67.54)
Yes, PPE was adequately allocated	422 (32.46)
**Did your PPE have a quality?**	-
No	94 (7.23)
Yes	1,206 (92.77)
**Was the shift schedule appropriately allocated?**	-
Yes, appropriate	842 (64.77)
No, excessive workload	458 (35.23)

**Table 2:** Prevalence of stress among healthcare personnel post-COVID-19 pandemic, northeast Thailand.

**Factors**	**Stress Level; N (%)**			
	**Mild**	**Moderate**	**Severe**	**Very Severe**
**Overall Stress**	555 (42.69)	544 (41.85)	86 (6.62)	115 (8.85)
**Personal Characteristics**	-	-	-	-
**Gender**	-	-	-	-
Male	168 (42.21)	159 (39.95)	35 (8.79)	36 (9.05)
Female	387 (42.90)	385 (42.68)	51 (5.65)	79 (8.76)
**Age**	-	-	-	-
< 30	132 (41.38)	132 (41.38)	33 (10.34)	22 (6.9)
30 - 39	226 (43.71)	212 (41.01)	26 (5.03)	53 (10.25)
40 - 49	151 (45.76)	142 (43.03)	17 (5.15)	20 (6.06)
≥ 50	46 (34.33)	58 (43.28)	10 (7.46)	20 (14.93)
**Status**	-	-	-	-
Single or devoted	179 (45.43)	171 (43.4)	28 (7.11)	16 (4.06)
Married	376 (41.5)	373 (41.17)	58 (6.4)	99 (10.93)
**Education**	-	-	-	-
Lower bachelor	113 (53.3)	76 (35.85)	13 (6.13)	10 (4.72)
Bachelor or higher	442 (40.63)	468 (43.01)	73 (6.71)	105 (9.65)
**Profession**	-	-	-	-
Other healthcare personnel	188 (51.37)	136 (37.16)	16 (4.37)	26 (7.1)
Physician and nurses	367 (39.29)	408 (43.68)	70 (7.49)	89 (9.53)
**Underlying diseases**	-	-	-	-
No	520 (44.44)	489 (41.79)	72 (6.15)	89 (7.61)
Yes	35 (26.92)	55 (42.31)	14 (10.77)	26 (20.0)
**Income**	-	-	-	-
< 404.50$	109 (49.77)	81 (36.99)	17 (7.76)	12 (5.48)
404.50 - 809.00$	201 (41.02)	193 (39.39)	41 (8.37)	55 (11.22)
> 809.00$	245 (41.46)	270 (45.69)	28 (4.74)	48 (8.12)
**Adequate income**	-	-	-	-
Inadequate	83 (44.15)	75 (39.89)	17 (9.04)	13 (6.91)
Adequate but no saving	129 (45.42)	128 (45.07)	8 (2.82)	19 (6.69)
Adequate and saving	343 (41.43)	341 (41.18)	61 (7.37)	83 (10.02)
**Workplace**	-	-	-	-
Primary care	125 (45.79)	125 (45.79)	10 (3.66)	13 (4.76)
Secondary or tertiary hospital	430 (41.87)	419 (40.8)	76 (7.4)	102 (9.93)
**Work experience**	-	-	-	-
< 10 years	269 (44.76)	226 (37.60)	50 (8.32)	56 (9.32)
≥ 10 years	286 (40.92)	318 (45.49)	36 (5.15)	59 (8.44)
**Working hours per week**	-	-	-	-
≤ 40 hours per week	171 (43.85)	158 (40.51)	22 (5.64)	39 (10.0)
> 40 hours per week	384 (42.20)	386 (42.42)	64 (7.03)	76 (8.35)
**Motivating factors**	-	-	-	-
**Number of family members**	-	-	-	-
< 5	187 (43.09)	187 (43.09)	43 (9.91)	17 (3.92)
≥ 5	368 (42.49)	357 (41.22)	43 (4.97)	98 (11.32)
**Had a grandchild who was < 5 years old**	-	-	-	-
No	430 (46.54)	380 (41.13)	52 (5.63)	62 (6.71)
Yes	125 (33.24)	164 (43.62)	34 (9.04)	53 (14.10)
**Lived with elderly**	-	-	-	-
No	140 (43.21)	141 (43.52)	25 (7.72)	18 (6.0)
Yes	415 (42.52)	403 (41.29)	61 (6.25)	97 (9.94)
**A family member had NCDs**	-	-	-	-
No	381 (45.79)	350 (42.07)	58 (6.97)	43 (5.17)
Yes	174 (37.18)	194 (41.45)	28 (5.98)	72 (15.38)
**Have you been quarantined because you had a risk of being infected with COVID-19?**	-	-	-	-
No	95 (48.47)	85 (43.37)	16 (8.16)	0 (0)
Yes	460 (41.67)	459 (41.58)	70 (6.34)	115 (9.0)
**Have you been infected with COVID-19?**	-	-	-	-
No	89 (42.18)	95 (45.02)	18 (8.53)	9 (4.27)
Yes	466 (42.79)	449 (41.23)	68 (6.24)	106 (9.73)
**Organizational administration**	-	-	-	-
**Did your organization support bonuses or allowances?**	-	-	-	-
No	20 (39.22)	15 (29.41)	7 (13.73)	9 (17.65)
Yes	535 (42.83)	529 (42.35)	79 (6.33)	106 (8.49)
**Did you get satisfactory bonuses or allowances?**	-	-	-	-
No	166 (37.56)	190 (42.99)	36 (8.14)	50 (11.31)
Yes	389 (45.34)	354 (41.26)	50 (5.83)	65 (8.0)
**Did your organization adequately allocate PPE to prevent COVID-19 infection?**	-	-	-	-
No, PPE was inadequately allocated.	348 (39.64)	388 (44.19)	61 (6.95)	81 (9.23)
Yes, PPE was adequately allocated	207 (49.05)	156 (36.97)	25 (5.92)	34 (8.06)
**Did your PPE have a quality?**	-	-	-	-
No	23 (24.47)	48 (51.06)	9 (9.57)	14 (14.89)
Yes	532 (44.11)	496 (41.13)	77 (6.38)	101 (8.37)
**Was the shift schedule appropriately allocated?**	-	-	-	-
Yes, appropriate	387 (45.96)	353 (41.92)	39 (4.63)	63 (7.48)
No, excessive workload	168 (36.68)	191 (41.7)	47 (10.26)	52 (11.35)

**Table 3:** Factors associated with stress among healthcare personnel post-COVID-19 pandemic in northeast Thailand were analyzed by simple and multinomial logistic regression analysis.

**Factors**	**Crude OR (95%CI)**	**P-value**	**Adj. OR (95%CI)**	**P-value**
**Personal Characteristics**	-	-	-	-
**Gender**	-	-	-	-
Male	1	-	-	-
Female	0.92 (0.73 to 1.15)	0.472	-	-
**Age**	-	-	-	-
< 30	1	0.022	-	0.046
30 - 39	0.92 (0.71 to 1.19)	-	0.76 (0.56 to 1.03)	-
40 - 49	0.79 (0.59 to 1.05)	-	0.72 (0.49 to 1.04)	-
≥ 50	1.42 (0.97 to 2.07)	-	1.12 (0.70 to 1.80)	-
**Status**	-	-	-	-
Single or devoted	1	0.023	1	0.03**0**
Married	1.13 (1.04 to 1.61)	-	1.36 (1.03 to 1.79)	-
**Education**	-	-	-	-
Lower bachelor	1	<0.001	1	0.002
Bachelor or higher	1.67 (1.25 to 2.21)	-	1.88 (1.25 to 2.80)	-
**Profession**	-	-	-	-
Other healthcare personnel	1	<0.001	1	0.026
Physician and nurses	1.61 (1.28 to 2.03)	-	1.36 (1.04 to 1.78)	-
**Underlying diseases**	-	-	-	-
No	1	<0.001	1	<0.001
Yes	2.49 (1.76 to 3.52)	-	3.30 (2.24 to 4.84)	-
**Income**	-	-	-	-
< 404.50$	1	0.029	1	<0.001
404.50 - 809.00$	1.51 (1.11 to 2.04)	-	0.97 (0.63 to 1.46)	-
> 809.00$	1.30 (0.97 to 1.74)	-	0.64 (0.41 to 0.99)	-
**Adequate income**	-	-	-	-
Inadequate	1	0.104	1	0.022
Adequate but no saving	0.86 (0.61 to 1.22)	-	0.69 (0.52 to 0.92)	-
Adequate and saving	1.13 (0.84 to 1.52)	-	1.05 (0.77 to 1.48)	-
**Workplace**	-	-	-	-
Primary care	1	0.022	1	<0.001
Secondary or tertiary hospital	1.34 (1.04 to 1.72)	-	1.89 (1.44 to 2.49)	-
**Work experience**	-	-	-	-
< 10 years	1	0.696	-	-
≥ 10 years	1.04 (0.85 to 1.28)	-	-	-
**Working hours per week**	-	-	-	-
≤ 40 hours per week	1	0.752	-	-
> 40 hours per week	1.04 (0.83 to 1.30)	-	-	-
**Motivating factors**	-	-	-	-
**Number of family members**	-	-	-	-
< 5	1	0.368	-	-
≥ 5	1.10 (0.89 to 1.37)	-	-	-
**Had a grandchild who was < 5 years old**	-	-	-	-
No	1	<0.001	1	<0.001
Yes	1.88 (1.50 to 2.36)	-	1.89 (1.48 to2.41)	-
**Lived with elderly**	-	-	-	-
No	1	0.437	-	-
Yes	1.10 (0.87 to 1.39)	-	-	-
**A family member had NCDs**	-	-	-	-
No	1	<0.001	1	<0.001
Yes	1.62 (1.30 to 2.00)	-	1.51 (1.21 to 1.89)	-
**Have you been quarantined because you had a risk of being infected with COVID-19?**				-
No	1	0.006	1	0.009
Yes	1.48 (1.12 to 1.97)	-	1.52 (1.11 to 2.07)	-
**Have you been infected with COVID-19?**	-	-	-	-
No	1	0.645	-	-
Yes	1.07 (0.81 to 1.40)	-	-	-
**Organizational administration**	-	-	-	-
**Did your organization support bonuses or allowances?**		-	-	-
No	1.63 (0.94 to 2.82)	-	2.51 (1.42 to 4.46)	-
Yes	1	0.082	1	0.002
**Did you get satisfactory bonuses or allowances?**	-	-	-	-
No	1.43 (1.15 to1.78)	<0.001	-	-
Yes	1	-	-	-
**Did your organization adequately allocate PPE to prevent COVID-19 infection?**				-
No, PPE was inadequately allocated.	1.39 (1.12 to 1.74)	-	-	-
Yes, PPE was adequately allocated	1	0.003	-	-
**Did your PPE have a quality?**	-	-	-	-
No	2.15 (1.46 to 3.16)	-	2.56 (1.71 to 3.82)	-
Yes	1	<0.001	1	<0.001
**Was the shift schedule appropriately allocated?**		-	-	-
Yes, appropriate	1	<0.001	1	0.005
No, excessive workload	1.59 (1.28 to 1.98)	-	1.40 (1.11 to 1.78)	-

## Data Availability

The data and material sources that support the findings of this study are available from the corresponding author [P.L] upon reasonable request.

## LIST OF ABBREVIATIONS

Adj. OR: = Adjusted Odd Ratio
95%CI: = 95%Confidence Interval
COVID-19: = Coronavirus disease 2019
Crude OR: = Crude Odd Ratio
NCDs: = Non-Communicable Diseases
PPE: = Personal Protective Equipment

## STANDARDS OF REPORTING

STROBE guidelines were followed.
